# A Review Article of the Reduce Errors in Medical Laboratories

**DOI:** 10.5539/gjhs.v7n1p46

**Published:** 2014-07-29

**Authors:** Zuhair M. Mohammedsaleh, Fayez Mohammedsaleh

**Affiliations:** 1 Faculty of Applied Medical Sciences, University Of Tabuk, Tabuk, Kingdom of Saudi Arabia; 2 Faculty of Medicine, University of Tabuk, Tabuk, Kingdom of Saudi Arabia

**Keywords:** medical laboratory errors, pathology, laboratory staff skills, clinical laboratory

## Abstract

The current article examines the modern practices of reducing errors in medical laboratories. The paper sought to examine the methods that different countries are applying to reduce errors in medical laboratories. In addition, the paper examines the relationship between inadequate training of laboratory personnel and error causation in medical laboratories. A total of 17 research articles have been reviewed. The paper has done a comparison of pathology laboratory practices in the US, Canada, the UK and Australia, regarding laboratory staff skills and error reduction. The paper finds out that; although some of the developed countries have employed advanced technology to reduce errors, there is still a great need to use sophisticated medical equipment to reduce errors. In addition, the levels of training for the medical technicians are still low. They are not equipped enough to reduce the errors to the required levels. The article recommends application of advanced technology in the reduction of errors, and training of technicians on the best practices to reduce errors.

## 1. Introduction

The risks involved in the health profession are not excluded in the medical laboratories. The risks can pose serious consequences to both the laboratory staffs and the patients. In some cases, they emanate from the wide range of mechanical, chemical, biological and environmental hazards that the laboratory practices involve (Buesa, 2007). However, the risks are also caused by the errors that occur in the laboratories. While some of the errors are caused by lack of adequate knowledge and skills among the medical laboratory personnel, others are caused by ineffective equipments used in the laboratories. There are universal, as well as national regulations that regulate the qualifications for entry into the medical laboratory profession in different countries.

Although the standards in many countries have been improved to address the problem of inadequate training, there is still a gap between the required and the current level of knowledge and skills for the clinical laboratory personnel (Zima, 2010). In addition, some medical laboratories have been able to implement the new modern technologies that are more effective in reducing errors. However, others, especially those that are in community hospitals, have not been able to implement them. The problems have contributed immensely to the numerous cases of errors that occur in clinical laboratories (Zima, 2010). The eventual impact of such errors is the poor quality of care that is provided to patients. The current article presents a review of the errors that occur in the medical laboratories and strategies for reducing them, through enhancing the knowledge and skills of the laboratory personnel. Prior to addressing the main topic, the article gives an overview of the certification and training levels of laboratory personnel in the US, as well as the UK laboratory system.

## 2. Certification and Training of Medical Laboratories Staff

Different countries have different certification requirements for clinical laboratories. In the US, the federal standards require all medical laboratories are certified under the 1988 Laboratory Improvement Amendments Act (CLIA). The certification given depends on the kind and complexity of testing taking place in the laboratory (Walz, 2013). Other bodies that inspect and set regulations to laboratory practices in the US include the Commission on Laboratory Accreditation, the Joint Commission and College of American Pathologists (Sciacovelli et al., 2010). The standards set in CLIA apply in all countries worldwide. CLIA standards define the roles of each laboratory staff and the required qualifications, depending on the level and complexity of responsibility.

However, Walz (2013) explains, the qualification requirements set by CLIA are considered inadequate by the laboratory professional community, and are viewed as having contributed to the high number of errors that have been occurring in the pathology laboratories. For instance, the staff qualification requirements set in CLIA standards for the moderate and high class laboratories are a high school diploma and additional training in the required field. The problem prompted the 2005 task force of the American Society for the Clinical Laboratory Sciences that explored the educational levels and the requirements of the laboratory staffs. The task force’s position paper was adopted in 2009. In response, the laboratory professional community has responded by setting their own qualification standards for different staff members working in medical laboratories.

**Figure 1:**
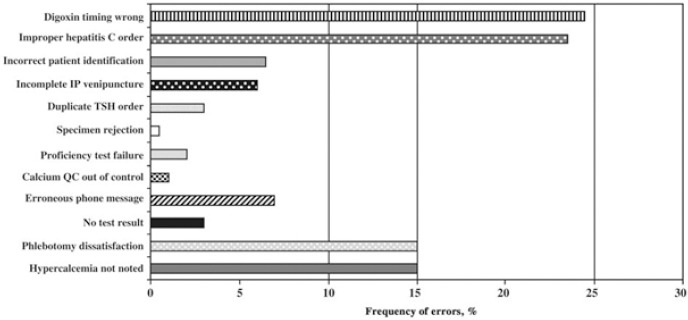
Laboratory error rate identified by the American College of Pathologists Source: Plebani (2006).

Despite the intervention of the laboratory professional community, the current standards set for laboratory workers such as laboratory attendants, assistants/technicians, medical technologists and consultants are still low. As Walz (2013) explains, lower level of standards has contributed to the low level of training among the different staff members working in medical laboratories. For instance, the pathology laboratory technicians are required to attend training programs offered by different organizations in the US and seek certification through agencies such as American Medical Technologists and National Credentialing Agency for Laboratory Personnel. However, the level of training is not well regulated, and the technicians may qualify after attending training programs for just seven months. As a result, they do not receive adequate training to enable them handle complex tests in clinical laboratories (Walz, 2013). The problem has contributed immensely to the increased number of errors in the pathology laboratories. Figure 1 in the presents the laboratory error rate identified by the American College of Pathologists.

## 3. Evaluation of the UK Laboratory System

In the UK, the Clinical Pathology Accreditation (CPA-UK) recognizes that a laboratory is an entity that should be held legally responsible (CPA-UK, 2007). In line with this, all the laboratories are required to operate as per the regulations of the CPA that govern laboratory operations. The law thus requires all personnel working in a medical laboratory to have adequate training, authority and resources in order to carry out their duties effectively. In addition, the law requires arrangements to be made to shield the laboratory from internal, external and financial pressures that may compromise results (CPA-UK, 2007). Further, the laboratories are required to make arrangements that would ensure that clients do not lose confidence in the services they provide.

The UK laboratories have a quality policy statement that identifies their scope of practice, standards, intention, commitment to good professional practice, commitment to legislation and a commitment to the welfare and safety of all the staff members, clients and visitors. In terms of personnel, the UK laboratories are headed by a laboratory director, just like in the US. The director is required to have consultative, educational, administrative and scientific skills. The policy also requires laboratories to have enough personnel to prevent errors that may occur as a result of work overload due to staff shortage. Adherence to the set policies and standards can help to reduce errors in the laboratories. However, medical laboratories in the UK face shortages of personnel in some areas. For instance, the number of trained medical technologists in clinical laboratories in the UK has been reducing over the last one decade. The problem has also been experienced recently in the US. The problem has been caused by a low level of entry into the profession and a high level exit. The fundamental causes of the problem are the lack of attractive remuneration and too much pressure, among others. Addressing such problems can help to attract more candidates into the profession and hence, face out the problem of shortage. Eventually, the move can help to reduce the number of errors occurring in the laboratory as a result of overload.

## 4. Laboratory Errors and Quality Enhancement

**Figure 2:**
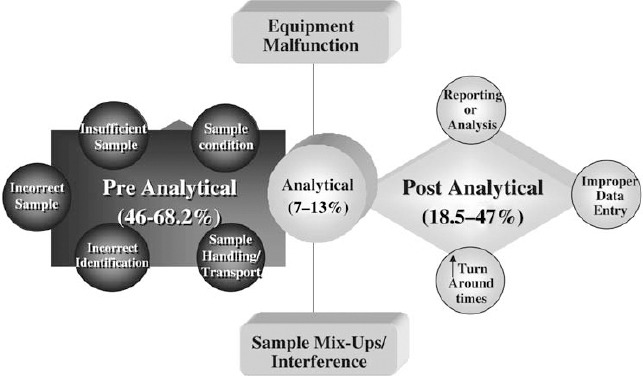
The rates and types of errors in the three stages of the laboratory process Source: Plebani (2006).

Karkalousos and Evangeloupos (2011) categorize errors depending on the stage of the testing process they occur. In other words, errors can occur in the pre-analytic stage, analytic stage or post-analytic stage (Karkalousos & Evangeloupos, 2011). The pre-analytical stage includes the procedures that are carried out before the analysis of the samples of the patients. Pre-Analytical errors include inappropriate specimen, the wrong anticoagulant, improper conservation method, mistakes in patient identification and inappropriate preparation of the patient (Karkalousos & Evangeloupos, 2011). The analytical errors occur during the analysis stage. According to Karkalousos and Evangeloupos (2011), most of the errors that occur in the analytical stage are caused by laboratory personnel. Examples of analytical errors include expired reagents, sampling system failure, analyzer failure and a timed-out calibration system (Karkalousos & Evangeloupos, 2011). The post-analytical errors include wrong matching, loss of the results and wrong copy of the results. Figure 2 in the appendix presents the rates and types of errors that occur in the three laboratory testing phases. The errors have significant implications because they affect the reproducibility, precision, accuracy and repeatability of results. Table 1 presents some effects of laboratory errors on patient care.

**Table 1:** The effect of laboratory errors on patient care

	Numberof errors	Effect on patientcare	Risk of inappropriate care
• Rossand Boone(70)	336	30	7
• Nutting et al. (71)	180	27	12
• Plebaniand Carraro (11)	189	26	6.4

Source: Plebani (2006).

Some laboratories have installed automated analyzers in an attempt to reduce errors or to generate credible laboratory results. The automated analyzers are capable of producing multiple results in a short time. As Karkalousos and Evangeloupos (2011) explain, this has been enabled through the incorporation of technology of robotics, computer science and analytical chemistry. However, as Karkalousos and Evangeloupos (2011) explain, statistical quality control is a bit difficult to monitor due to the large number of samples that are taken for testing. In addition, the wide range of varying diagnosis tests conducted in laboratories makes it difficult to use automated analyzers without making errors. As such, automated analyzers are hardly effective in reducing errors in laboratories.

A study conducted by Layfield and Anderson (2010) revealed that 75% of the errors, in a study of a span of 18 months, involved wrong patient labelling. 24% of the errors involved the site that was being labelled. Layfield and Anderson (2010) note that, even though many laboratories have focused on the errors described by the Institute of Medicine’s report of 1999, specimen labelling errors have not been adequately addressed. According to Layfield and Anderson (2010), the identification errors are also prevalent in the laboratories, and only a few studies have documented them. The errors lead to a significant harm and inconveniences in patient care. Some of the techniques used to identify labelling errors have often under-estimated the frequency of occurrence of errors, making it difficult to record all the errors that occur in the laboratories (Layfield & Anderson, 2010). However, the pathologists, laboratory staff and clinicians are in a better position to identify labelling and other types of errors. Since expertise is needed in such detection, inadequate skills among the laboratory personnel are likely to increase the frequency of errors in the laboratories.

According to Hammerling (2012), the difficulties in translating new knowledge to practice and implementing new technology are among the factors inhibiting effective reduction of errors in medical laboratories. Numerous researchers have made recommendations on how to reduce errors in medical laboratories. However, lack of adequate knowledge and training among the laboratory staffs makes it difficult to achieve the improvements. Precisely, many staff members in the medical laboratories are hardly updated with the new knowledge and ways of implementing the advanced, most effective equipments. In addition, some medical laboratories have not been able to acquire a modern, effective equipments. As such, Hammerling (2012) suggests that the incorporation of new technology in the medical laboratory can help to reduce the errors. In addition, Hammerling (2012) suggests that adequate skills among laboratory staff members can help to reduce documentation errors. Dintzis et al. (2011) highlights the importance of communication in reducing cases of errors in medical laboratories. According to Dintzis et al. (2011), effective communication between laboratory staff members and other health professionals such as doctors and nurses can help to determine the sources of errors as early as possible and to take the necessary action to avoid them. Lippi and Plebani (2009) also highlight the importance of reporting cases of errors in order to establish ways of rectification.

## 5. Modern Systems to Reduce Errors

The modern methods of laboratory error reduction have been applied at the pre-analytical, analytical, and post-analytical stages of specimen processing in the laboratories (Plebani, 2006). However, the pre- and post-analytical stages have been found to be more prone to errors than the analysis phases. The main cause of the problem is that some phases are not in direct control of the laboratory personnel. Continuous monitoring and educational initiatives have highly contributed to the reduction of the errors in the laboratories. Plebani (2006) asserts that between 1999 and 2000, inter-laboratory Q-Tracks improvement programs showed that errors reduced from 7.4 to 3 percent due to continuous monitoring and educational initiatives. Interdepartmental cooperation has also been identified as a measure that has contributed to the reduction of laboratory errors (Plebani, 2006).

The automated analyzers used in the pre-analytical stage have also helped to reduce laboratory errors. The hazards and errors at this stage have further been reduced through the introduction of analytical robotic workstations that are automated (Plebani, 2006). The automatic workstations have helped to reduce errors of labelling, sorting, routing and pour-off. The analytical stage errors have also been significantly reduced through technological advancement and standardization (Plebani, 2006). For example, a high level of accuracy has been achieved in the testing of blood products for infectious agents. Plebani (2006) observes that nucleic acid testing has reduced contamination rates in blood testing from 1 in 100 units to 1 in 1.8 million units. However, interference with immunoassays has led to serious errors.

According to Lippi, Plebani and Šimundić (2010), efforts tailored to reduce medical laboratory errors have been successful in improving patient care in many cases. Lippi et al. (2010) propose the need to address the fundamental causes of the errors, rather than individuals. In addition, Lippi et al. (2010) highlight the importance of implementing effective modern technology and supporting decision-making processes in reducing cases of clinical laboratory errors. According to Da Rin (2009), accountability should also be improved using performance and outcome measures. DA Rin (2009), cite heterogeneity in the definition of laboratory errors as a hindrance to good, standardized method of reducing laboratory errors. Da Rin (2009), also emphasizes that the increase in the reduction of laboratory errors has a connection to the lack of adequate training of medical laboratory personnel. In addition, Da Rin (2009), proposes that rules should be adopted to define the allowable errors in order to reduce the errors that arise due to quackery. Proficiency testing programs and external assessment schemes have been found to be effective in reducing laboratory errors. These schemes have also been effective in detecting the sources of errors. According to Da Rin (2009), team working and departmental corporation can also help to reduce laboratory errors.

## 6. Reducing Errors in Australia and Canada and Successful Examples

In Australia, several strategies have been adopted to reduce laboratory errors. Rin (2009) observes that a comprehensive five-step interrelated plan has been devised to reduce errors in Australian laboratories. These steps include developing written procedures that are clear, enhancing healthcare professional training, monitoring laboratory indicators, automating laboratory functions, and improving communication among the healthcare providers, and also between the care providers and the patients. In Australia, the automation of pre-analytic workstations in the prevention of errors is an example of a successful intervention. According to Rin (2009), the incorporation of automated robotic workstations has greatly reduced the human errors that occurred through human processing. In addition, the automated workstations improved the handling of specimens. The introduction of workstations was also been accompanied by the setting of specific quality goals to ensure that errors in the laboratories are reduced.

A case of success in reducing laboratory errors has also been documented in regard to San Bassanio hospital. Some of the steps that the management implemented to improve the reduction of laboratory errors are mentioned here. The management installed a wireless network in the hospitals to enhance easy access to images and medical records (Rin, 2009). Other clinical applications have also been employed to ensure that recording of patient data is easily done, even at the bedside. Secondly, computerized order entry systems with highly effective laptops were introduced (Rin, 2009). The improvement has enabled the laboratory personnel to carry out tests and radiology at the patient’s bedside. Requesting of tests can be done online, hence reducing the errors that result from documentation. Also, automated sample-labelling systems have been introduced in order to reduce labelling errors. The systems are also accompanied by test tube kits that contain the spaces that the laboratory staff should just fill. Another improvement is the introduction of barcode ID wristbands for both the outpatients and inpatients. The barcode has enabled the system succeed in the reduction of identification errors (Rin, 2009). The other improvement procedure in the reduction of errors is the standardization of collection procedures. Rin (2009) suggests that nurses should receive training in order to make this implementation process a success. Finally, the system has been enhanced through the incorporation of wireless internet to enhance communication between the different departments of the hospital that depend on the laboratory. Leape (2009) asserts that Canada has also recorded progress in incorporating these measures to ensure patient safety.

## 7. Conclusion

In conclusion, the review has found that laboratory errors are prevalent and that they are facilitated by defects in the equipment and mistakes by the laboratory staffs. Many of the errors are as a result of adequate training and shortage of laboratory staff members. The article has also established that laboratory directors and the pathologists are not satisfied with the training system for the laboratory staff. With the demand for the application of new technology and knowledge in medical laboratories, it warrants that laboratory staffs receive high level education. A comparison of literature shows that training of laboratory staffs has a direct bearing on the reduction of errors in the laboratories. In regard to reduction of errors using modern techniques, some laboratories in the United States, Australia, UK and Canada have adopted new technologies to effect the same. They include the incorporation of workstations, wireless network, automated labelling and standardization of collection procedures. Most of the studies consulted agree that training is an important aspect in reducing medical laboratory errors. Most academic studies need to be conducted in this crucial area in order to ascertain the role of adequate skills in reducing laboratory errors.
